# Fluorine–hydrogen inter­actions observed in a helix structure having an orn-free gramicidin S sequence incorporating 4-*trans*-fluoro­proline

**DOI:** 10.1107/S2056989025002592

**Published:** 2025-03-27

**Authors:** Asano Akiko, Mizuki Sakata, Kato Takuma, Mitsunobu Doi

**Affiliations:** a Osaka Medical and Pharmaceutical University, 4-20-1 Nasahara, Takatsuki, Osaka 569-1094, Japan; University of Hyogo, Japan

**Keywords:** crystal structure, fluorine-aromatic inter­action, gramicidin s, helix, fluoro­proline

## Abstract

The deca­peptide Boc-(d-Phe-tFPro-Val-Leu-Leu)_2_—OMe (**1**) (Boc is *tert*-but­oxy­carbonyl, tFPro is 4-*trans*-fluoro-l-proline d-Phe is d-phenyl­alanine, Val is valine and Leu is leucine) crystallized in a methanol-solvated form (C_68_H_104_F_2_N_10_O_13_·CH_4_O). Peptide **1** has a sequence similar to gramicidin S (GS) incorporating tFPro. GS is a cyclic peptide, with the d-Phe-Pro unit known as a strong β-turn inducer in previous studies. Thus, it was initially assumed that **1** would bend at the d-Phe6-tFPro7 position, potentially forming a sheet-like structure. However, the structure of **1** was a helix, a surprising finding in GS-related structural studies. A factor enabling this helical formation could be the fluorine–H inter­actions between tFPro and the aromatic rings of d-Phe residues.

## Abstract 2

Decapeptide having a gramicidin S sequence forms a helix structure supported by a fluorine–H inter­action.

## 1. Chemical context

Gramicidin S (GS) is a cyclic deca­peptide [cyclo­(Val-Orn-Leu-d-Phe-Pro)_2_] known for forming β-sheets and turns (Hodgkin & Oughton, 1957 ▸; Schmidt *et al.*, 1957 ▸). The Orn residues in GS contribute to its amphiphilicity, but the amino­propyl group causes high flexibility, hindering structural homogeneity (Asano & Doi, 2019 ▸). Previously reported Orn-free GS (LGS) mitigates this issue, providing an excellent scaffold for studying sheet and turn structures (Asano *et al.*, 2019 ▸; Asano *et al.*, 2021 ▸). Recently, we reported the structures of three LGS derivatives containing fluorinated proline (Asano *et al.*, 2023 ▸). During the synthesis, several linear deca­peptides were obtained before cyclization. One such derivative, Boc-(d-Phe-tFPro-Val-Leu-Leu)_2_-OMe (**1**), includes 4-*trans*-fluoro­proline (tFPro). Given the historical association of GS structures with turns and sheets (Balasubramanian, 1967 ▸; Tishchenko *et al.*, 1997 ▸; Doi *et al.*, 2001 ▸; Llamas-Saiz *et al.*, 2007 ▸), we anti­cipated that peptide **1** might bend at the central d-Phe-Pro moiety or form an anti­parallel sheet structure.
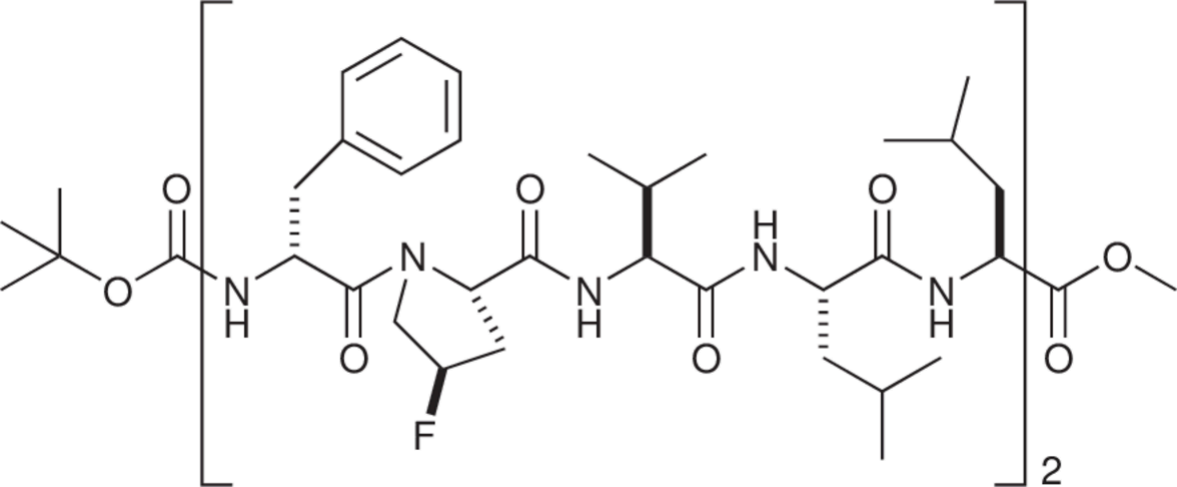


## 2. Structural commentary

**Figure 1:**
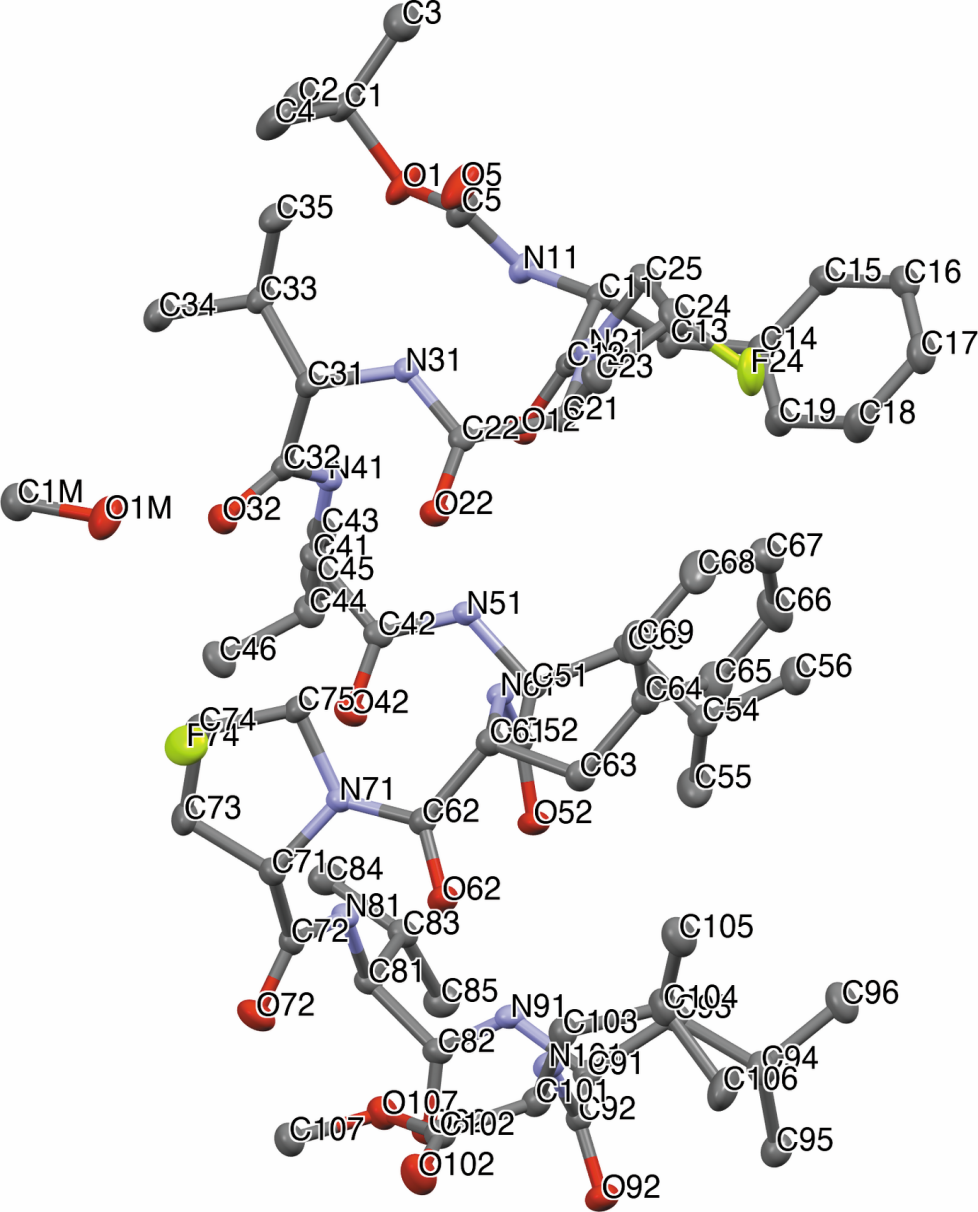
Peptide **1**, with displacement drawn at the 50% probability level.

**Figure 2:**
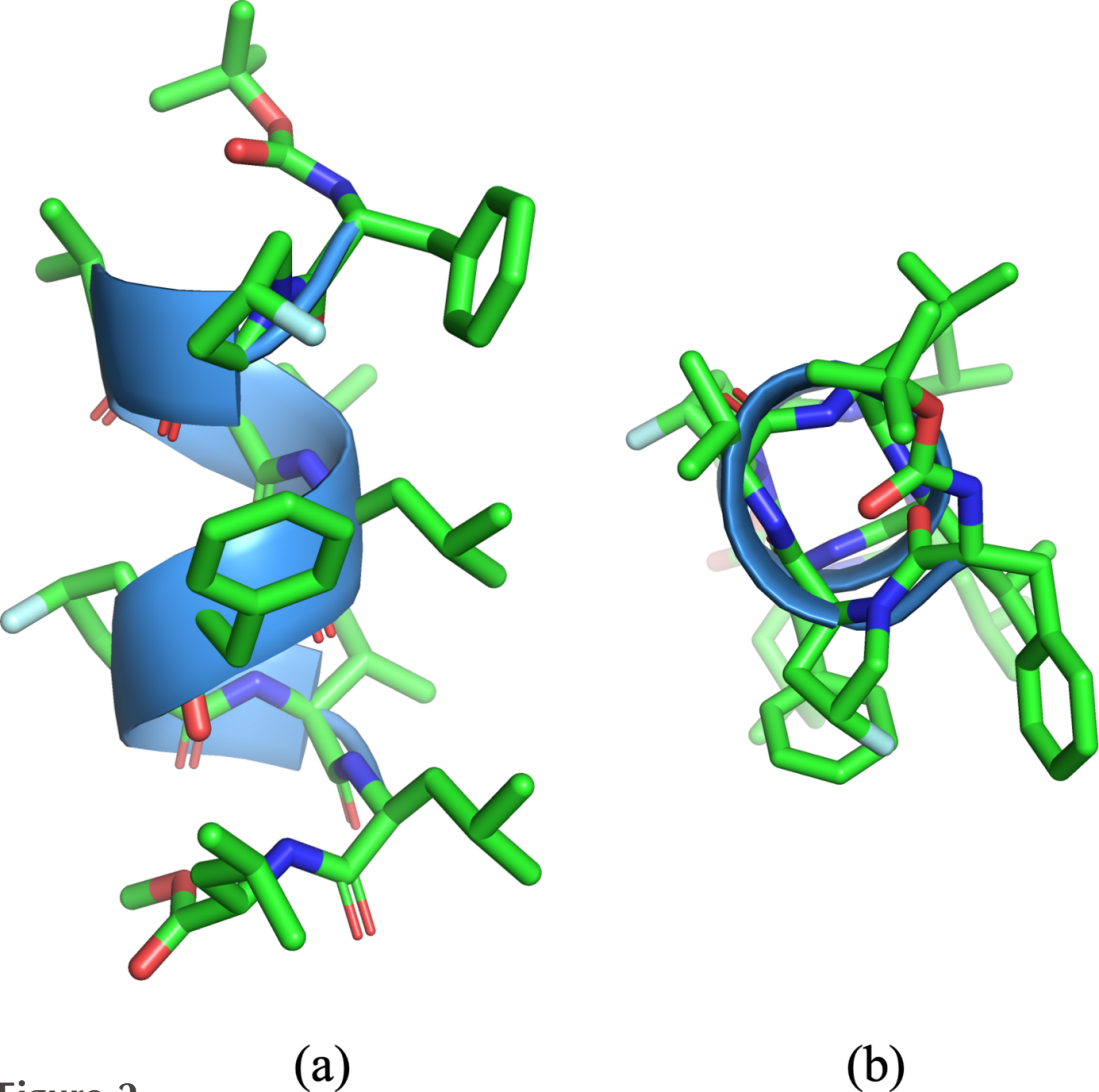
Ribbon models to enhance the helical structure. Projection views to (a) the side and (b) the top of the helix. Hydrogen atoms and solvent mol­ecule are omitted for clarity.

Fig. 1 ▸ shows that peptide **1** forms a helix structure. To enhance the clarity of the helical structure, a ribbon model is presented in Fig. 2 ▸. It is well known that gramicidin A (GA) containing d-amino acids forms the helix penetrating cell membranes (Hawkes *et al.*, 1987 ▸; Reddy *et al.*, 2018 ▸). It is quite different from the present helix. The torsion angles (Table 1 ▸) show the differences from the standard (φ,ψ) angles of the α-helix at the terminal residues (d-Phe1 and Leu10), and also at φ of Leu4 [−90.6 (2)°] and tFPro7 [169.2 (1)°]. Although the Pro residue is known as a helix breaker (Rohl *et al.*, 1996 ▸), tFPro7 leads to only small distortions to the helix structure. Such a case resembles Buforin-II having a Pro hinge, which forms the amphipathic helix (Yi, *et al.*, 1996 ▸; Park *et al.*, 2000 ▸). Moreover, it is notable that the (φ,ψ) angles of 6th residue is standard in α-helix, regardless of d-amino acid. The d-Phe-Pro moiety has been a pivot of turn in past studies of GS derivatives, but the central d-Phe6-tFPro7 remained a helix.

**Figure 3:**
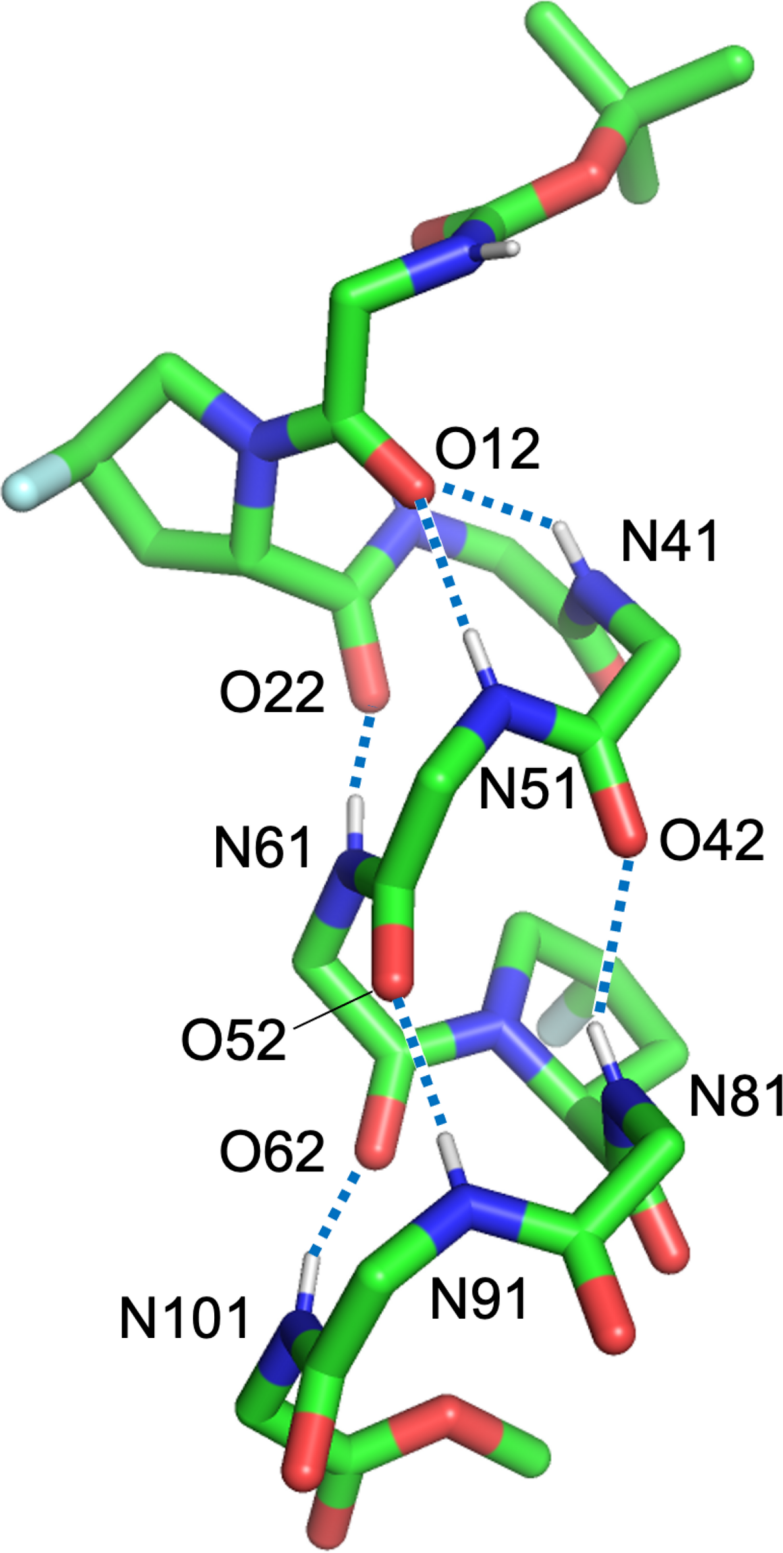
Hydrogen-bond networks of the helix. The backbone and hydrogen atoms involved in hydrogen bonds are drawn.

Hydrogen-bonded networks relating the backbone are shown in Fig. 3 ▸ and Table 2 ▸. Hydrogen bonds are formed between C=O and the H—N group of four residues upstream (i+4 and i), and thirteen atoms are involved in the ring formed by the hydrogen bond in the α-helix. Five hydrogen bonds, namely N51⋯O12, N61⋯O22, N81⋯O42, N91⋯O52 and N101⋯O62, involve thirteen atoms. However, atom N41 inter­acts with O12 [N41⋯O12 = 2.968 (2) Å] forming a ten-atom ring (H41–N41–C32–C31–N31–C22–C21–N21–C12–O12), which is characteristic for a 3_10_-helix. The features of the two helix types coexist through O12. It would relate to the inter­action between the fluorine atom of tFPro2 and the phenyl ring of d-Phe1.

**Figure 4:**
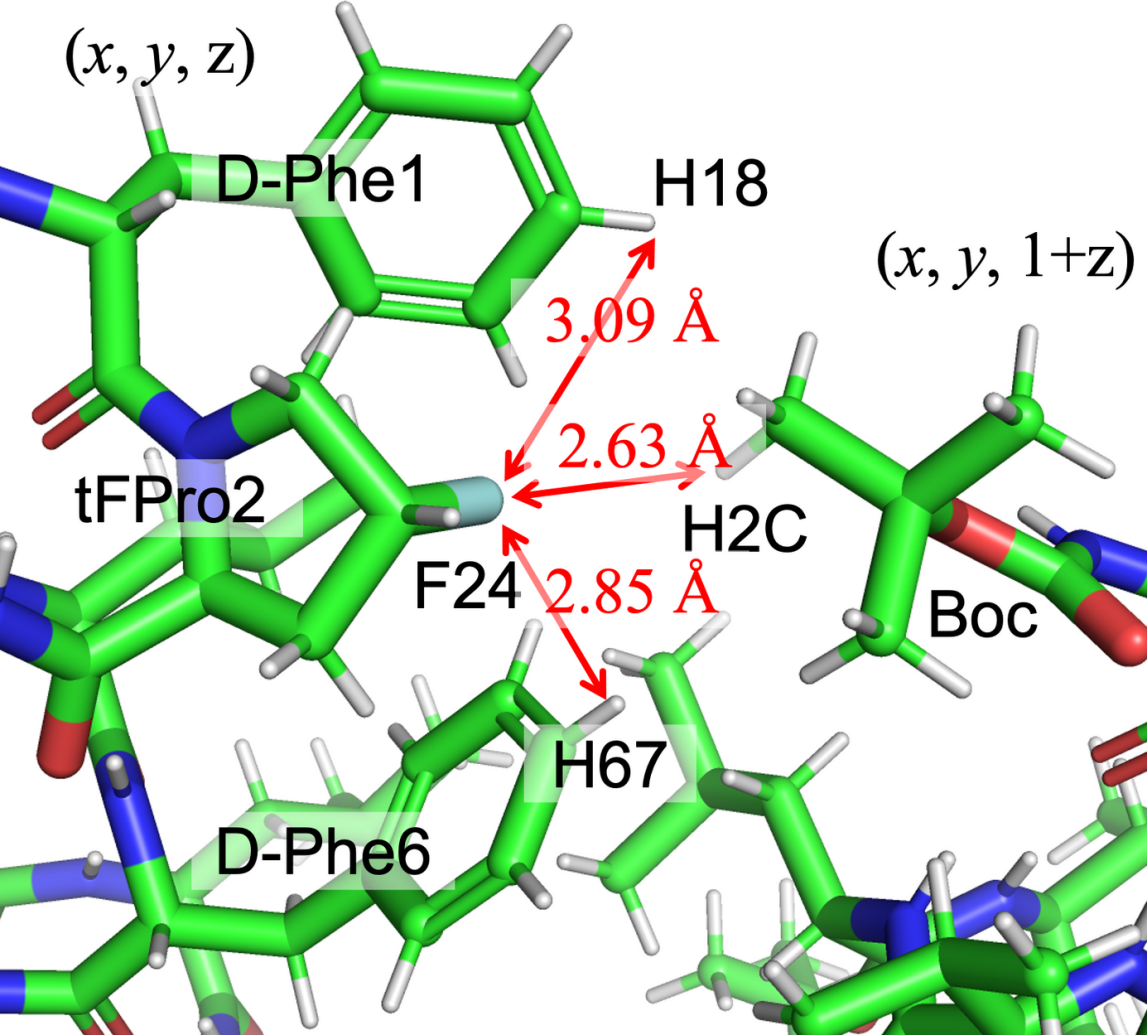
F⋯H inter­actions around atom F24. F24⋯H18(d-Phe1), F24⋯H26(d-Phe6) and F24⋯H2*C*[Boc translated by (*x*, *y*, *z* + 1)].

The structure around the F24 atom is shown in Fig. 4 ▸, where the phenyl rings of d-Phe1 and d-Phe6 flank the F24 atom. While F24 is not positioned directly above the phenyl ring, it is close to the hydrogen atoms, with distances of F24⋯H18 = 3.09 Å and F24⋯H67 = 2.85 Å. Given that F⋯H inter­actions typically occur at < 2.90 Å (Thalladi, *et al.*,1998 ▸), it can be assumed that F24 engages in F⋯H inter­actions with both phenyl rings. This inter­action likely contributes to the cohesion of the helical structure, despite the presence of two d-amino acids in the peptide. Furthermore, the *trans* configuration of the fluorine atom in tFPro2 may facilitate its proximity to the phenyl rings within the helix

The puckering parameters of Pro are listed in Table 3 ▸. The signs of (χ1, χ2, χ3, χ4,θ) were approximately (−, +, −, +, ∼0) in both tFPro, which exhibits the Cγ-exo form (up form). In the GS analogues, the ‘down’ form (+, −, +, −, ∼0) is stable and often observed. The *trans* configuration of fluorine atom forces the puckering ‘up’ in **1**. A similar ‘up’ form has also been observed in tFPro residues of cyclic GS analogue (Asano *et al.*, 2023 ▸).

## 3. Supra­molecular features

**Figure 5:**
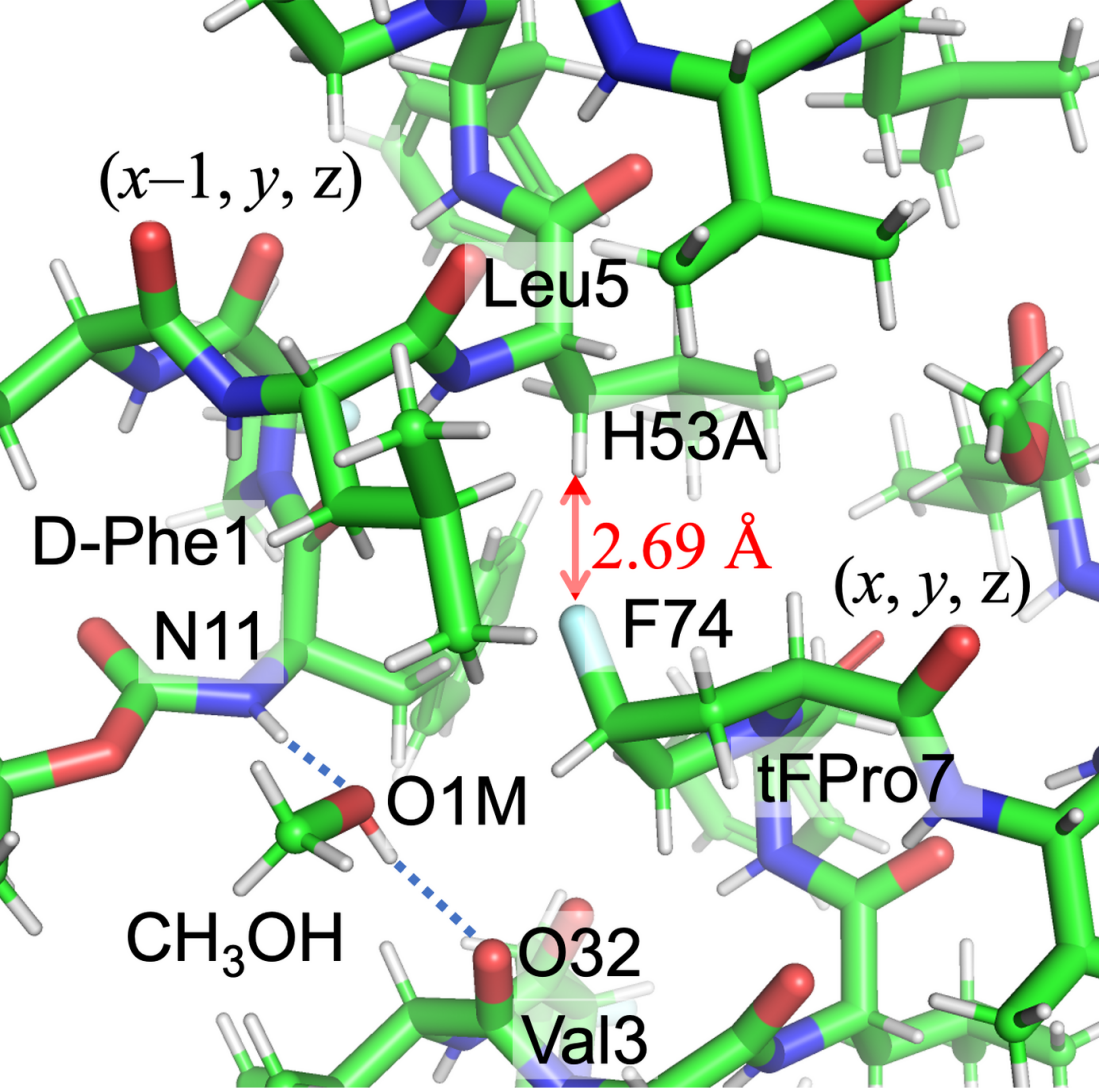
Inter­molecular inter­actions. Hydrogen bonds through the methanol mol­ecule bridge the original and its (*x* − 1, *y*, *z*)-translated mol­ecule. An F⋯H inter­action is formed, F74 ⋯H53*A* (Leu5).

**Figure 6:**
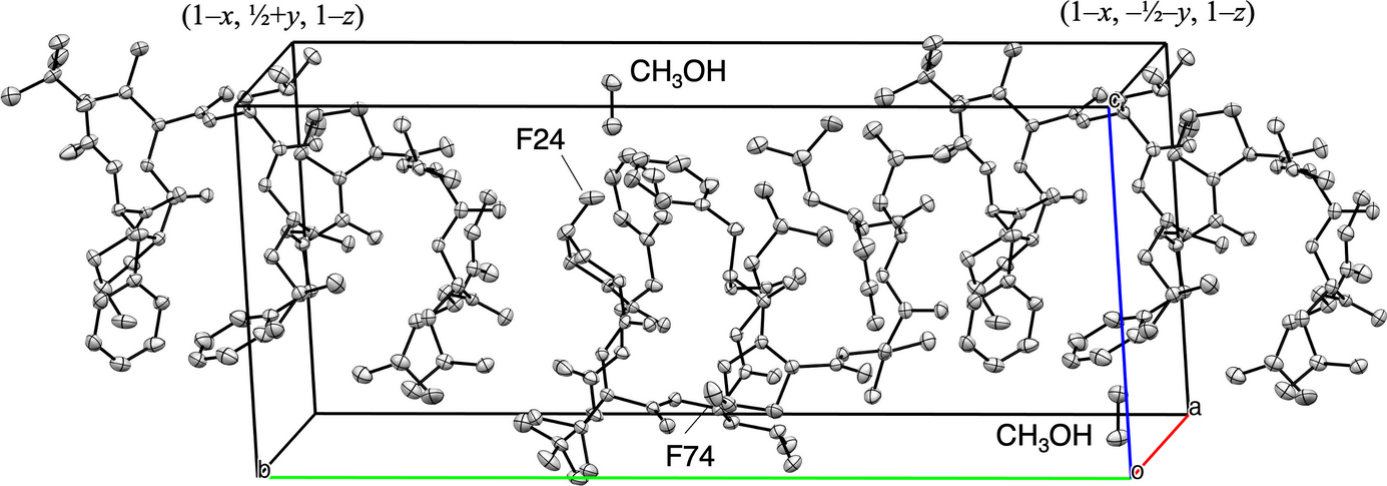
Head-to-tail arrangement formed along *b*-axis.

Fig. 5 ▸ illustrates the inter­actions between the original mol­ecule and its symmetry-related counterpart translated by (*x* − 1, *y*, *z*). The methanol mol­ecule (O1*M*) acts as a bridge between adjacent peptides, forming hydrogen bonds with O32 of Val3 [O1*M*⋯O32(Val3) = 2.703 (2) Å] and N1 of d-Phe1 [N1(d-Phe1)⋯O1*M* = 2.880 (2) Å]. An inter­molecular F⋯H inter­action is observed between F74 and H53*A* of the Leu5 methyl­ene group. Additionally, another F⋯H inter­action occurs between the original mol­ecule and its (*x*, *y*, *z* + 1)-translated mol­ecule, involving F24 and H2*C* from the Boc methyl group [F24⋯H2*C*(Boc) = 2.63 Å]. Since the methyl group can rotate, any of its three hydrogen atoms could potentially inter­act with the F24 atom. These inter­actions contribute to the expansion along the *a*-axis direction. Furthermore, the peptides align along the *b*-axis direction in a head-to-tail arrangement (Fig. 6 ▸).

## 4. Database survey

A search of the the CSD (WebCSD accessed February 2025; Groom *et al.*, 2016 ▸) and FIZ Karlsruhe’s free service indicates 43 records, but all records show the cyclic peptide. A search for the sequence *D*–Phe–Pro–Val–Leu–Leu using Google Scholar gave one hit, which is also the cyclic peptide. Compound **1** is unprecedented.

## 5. Synthesis and crystallization

Compound **1** was synthesized by a conventional liquid method using Boc (*tert*-but­oxy­carbon­yl) protection and purified by silicagel column chromatography. Crystals of **1** were grown in aqueous methanol (> 80%) solution.

## 6. Refinement

Crystal data, data collection and structure refinement details are summarized in Table 4 ▸. All H atoms were located in difference maps and were treated as riding in geometrically idealized positions with constrained distances set to 0.93 Å (C*sp*^2^—H), 0.98 Å (*R*_3_—CH), 0.97 Å (*R*_2_—CH_2_), 0.96 Å (*R*—CH_3_), 0.82 Å (*R*—OH) and 0.86 Å (*N*sp_2_—H). *U*_iso_(H) parameters were set to either 1.2 or 1.5 (methyl and hy­droxy groups) time those of the attached atom.

## Supplementary Material

Crystal structure: contains datablock(s) global, I. DOI: 10.1107/S2056989025002592/ox2013sup1.cif

Structure factors: contains datablock(s) I. DOI: 10.1107/S2056989025002592/ox2013Isup4.hkl

CCDC reference: 2432921

Additional supporting information:  crystallographic information; 3D view; checkCIF report

## Figures and Tables

**Table 1:** Backbone torsion angles (°) The values deviate from the standard α-helix (φ, ψ) = (−60°, −45°).

	i		i+5	
Residue	φ	ψ	φ	ψ
D-Phe1,6	75.0 (2)*	−129.2 (1)*	−52.2 (2)	−55.5 (2)
tFPro2,7	−56.4 (2)	−31.7 (2)	169.2 (1)*	−24.8 (2)
Val3,8	−50.5 (2)	−42.3 (2)	−73.6 (2)	−48.2 (2)
Leu4,9	−90.6 (2)*	−48.8 (2)	−89.0 (2)*	−44.4 (2)
Leu5,10	−64.6 (2)	−39.0 (2)	−93.0 (2)*	−8.3 (2)*

**Table 2:** Hydrogen-bond geometry (Å, °)

*D*—H⋯*A*	*D*—H	H⋯*A*	*D*⋯*A*	*D*—H⋯*A*
N41—H41⋯O12	0.88	2.29	2.968 (2)	134
N51—H51⋯O12	0.88	1.95	2.828 (2)	179
N61—H61⋯O22	0.88	2.09	2.907 (2)	154
N81—H81⋯O42	0.88	2.34	3.132 (2)	149
N91—H91⋯O52	0.88	2.05	2.913 (2)	168
N101—H101⋯O62	0.88	2.16	2.924 (2)	144
O1*M*—H1*M*⋯O32	0.84	1.88	2.703 (2)	164
N11—H11⋯O1*M*^i^	0.88	2.01	2.880 (2)	170

Symmetry code: (i) 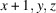.

**Table 3:** Puckering parameters (Å, °) of the pyrrolidine rings *Q*(2) and φ_2_ are defined by Cremer & Pople (1975 ▸) and calculated by *PLATON* (Spek, 2009 ▸).

Residue	*Q*(2)	φ_2_	χ_1_	χ_2_	χ_3_	χ_4_	θ
tFPro2	0.351 (2)	275.8 (3)	−26.6 (2)	36.5 (2)	−31.5 (2)	14.9 (2)	7.2 (2)
tFPro7	0.382 (2)	275.4 (2)	−29.1 (2)	39.6 (2)	−34.0 (2)	16.0 (2)	8.1 (2)

**Table 4:** Experimental details

Crystal data	
Chemical formula	C_68_H_104_F_2_N_10_O_13_·CH_4_O
*M*_r_	1339.65
Crystal system, space group	Monoclinic, *P*2_1_
Temperature (K)	100
*a*, *b*, *c* (Å)	10.1248 (1), 28.7263 (1), 12.6378 (1)
β (°)	96.484 (1)
*V* (Å^3^)	3652.17 (5)
*Z*	2
Radiation type	Cu *K*α
μ (mm^−1^)	0.73
Crystal size (mm)	0.20 × 0.20 × 0.15

Data collection	
Diffractometer	XtaLAB AFC12 (RINC): Kappa single
Absorption correction	Multi-scan (*CrysAlis PRO*; Rigaku OD, 2015 ▸)
*T*_min_, *T*_max_	0.902, 1.000
No. of measured, independent and observed [*I* > 2σ(*I*)] reflections	66224, 14120, 14063
*R*_int_	0.016
(sin θ/λ)_max_ (Å^−1^)	0.622

Refinement	
*R*[*F*^2^ > 2σ(*F*^2^)], *wR*(*F*^2^), *S*	0.025, 0.067, 1.02
No. of reflections	14120
No. of parameters	858
No. of restraints	1
H-atom treatment	H-atom parameters constrained
Δρ_max_, Δρ_min_ (e Å^−3^)	0.17, −0.21
Absolute structure	Flack *x* determined using 6582 quotients [(*I*^+^)−(*I*^−^)]/[(*I*^+^)+(*I*^−^)] (Parsons *et al.*, 2013 ▸)
Absolute structure parameter	−0.003 (16)

Computer programs: *CrysAlis PRO* (Rigaku OD, 2015 ▸), *SHELXT* (Sheldrick, 2015*a* ▸), *SHELXL2018/3* (Sheldrick, 2015*b* ▸), *Mercury* (Macrae *et al.*, 2020 ▸ and *pyMOL* (DeLano, 2002 ▸).

## supplementary crystallographic information

### Boc-(D-Phe-tFPro-Val-Leu-Leu)2-OMe Crystal data

**Table:**

C_68_H_104_F_2_N_10_O_13_·CH_4_O	*F*(000) = 1444
*M**_r_* = 1339.65	*D*_x_ = 1.218 Mg m^−^^3^
Monoclinic, *P*2_1_	Cu *K*α radiation, λ = 1.54184 Å
*a* = 10.1248 (1) Å	Cell parameters from 50791 reflections
*b* = 28.7263 (1) Å	θ = 4.4–73.5°
*c* = 12.6378 (1) Å	µ = 0.73 mm^−^^1^
β = 96.484 (1)°	*T* = 100 K
*V* = 3652.17 (5) Å^3^	Recutangular, colorless
*Z* = 2	0.20 × 0.20 × 0.15 mm

### Boc-(D-Phe-tFPro-Val-Leu-Leu)2-OMe Data collection

**Table:**

XtaLAB AFC12 (RINC): Kappa single diffractometer	14120 independent reflections
Radiation source: Rotating-anode X-ray tube	14063 reflections with *I* > 2σ(*I*)
Detector resolution: 5.8140 pixels mm^-1^	*R*_int_ = 0.016
multi–scan	θ_max_ = 73.7°, θ_min_ = 3.5°
Absorption correction: multi-scan (CrysAlisPro; Rigaku OD, 2015)	*h* = −12→12
*T*_min_ = 0.902, *T*_max_ = 1.000	*k* = −34→34
66224 measured reflections	*l* = −15→15

### Boc-(D-Phe-tFPro-Val-Leu-Leu)2-OMe Refinement

**Table:**

Refinement on *F*^2^	Hydrogen site location: inferred from neighbouring sites
Least-squares matrix: full	H-atom parameters constrained
*R*[*F*^2^ > 2σ(*F*^2^)] = 0.025	*w* = 1/[σ^2^(*F*_o_^2^) + (0.0437*P*)^2^ + 0.5472*P*] where *P* = (*F*_o_^2^ + 2*F*_c_^2^)/3
*wR*(*F*^2^) = 0.067	(Δ/σ)_max_ = 0.001
*S* = 1.02	Δρ_max_ = 0.17 e Å^−^^3^
14120 reflections	Δρ_min_ = −0.21 e Å^−^^3^
858 parameters	Absolute structure: Flack *x* determined using 6582 quotients [(*I*^+^)-(*I*^-^)]/[(*I*^+^)+(*I*^-^)] (Parsons *et al.*, 2013)
1 restraint	Absolute structure parameter: −0.003 (16)

### Boc-(D-Phe-tFPro-Val-Leu-Leu)2-OMe Special details

**Table:**

Geometry. All esds (except the esd in the dihedral angle between two l.s. planes) are estimated using the full covariance matrix. The cell esds are taken into account individually in the estimation of esds in distances, angles and torsion angles; correlations between esds in cell parameters are only used when they are defined by crystal symmetry. An approximate (isotropic) treatment of cell esds is used for estimating esds involving l.s. planes.

### Boc-(D-Phe-tFPro-Val-Leu-Leu)2-OMe Fractional atomic coordinates and isotropic or equivalent isotropic displacement parameters (Å^2^)

**Table:**

	*x*	*y*	*z*	*U*_iso_*/*U*_eq_	
O1M	0.10896 (13)	0.57492 (5)	0.04468 (10)	0.0321 (3)	
H1M	0.192244	0.573355	0.055394	0.048*	
C1M	0.0683 (2)	0.57447 (7)	−0.06624 (15)	0.0329 (4)	
H1M1	−0.028901	0.572951	−0.078656	0.049*	
H1M2	0.106412	0.547249	−0.098354	0.049*	
H1M3	0.099285	0.602902	−0.098497	0.049*	
C1	0.89151 (18)	0.71439 (6)	−0.06208 (13)	0.0264 (4)	
O1	0.93266 (14)	0.67532 (5)	0.00979 (9)	0.0306 (3)	
C2	0.9498 (2)	0.70048 (8)	−0.16328 (14)	0.0343 (4)	
H2A	0.928416	0.724329	−0.217970	0.051*	
H2B	1.046552	0.697558	−0.148214	0.051*	
H2C	0.912045	0.670577	−0.188838	0.051*	
C3	0.9547 (2)	0.75919 (7)	−0.01717 (17)	0.0370 (4)	
H3A	0.927131	0.785076	−0.065147	0.056*	
H3B	0.926067	0.765227	0.053074	0.056*	
H3C	1.051705	0.756172	−0.010456	0.056*	
C4	0.7419 (2)	0.71763 (8)	−0.08178 (17)	0.0381 (5)	
H4A	0.716866	0.743795	−0.129668	0.057*	
H4B	0.706072	0.688671	−0.114481	0.057*	
H4C	0.705519	0.722541	−0.013992	0.057*	
C5	0.90994 (18)	0.67612 (7)	0.11290 (13)	0.0260 (3)	
O5	0.83288 (16)	0.70154 (6)	0.15264 (11)	0.0400 (4)	
N11	0.98566 (13)	0.64363 (5)	0.16738 (10)	0.0201 (3)	
H11	1.032965	0.624054	0.133581	0.024*	
C11	0.98855 (15)	0.64121 (5)	0.28205 (12)	0.0174 (3)	
H11A	0.998605	0.673258	0.312779	0.021*	
C12	0.85924 (15)	0.61941 (5)	0.31143 (11)	0.0158 (3)	
O12	0.82044 (11)	0.58219 (4)	0.26970 (9)	0.0194 (2)	
C13	1.10720 (15)	0.61089 (6)	0.32765 (12)	0.0215 (3)	
H13A	1.086478	0.577716	0.312461	0.026*	
H13B	1.186409	0.619193	0.292328	0.026*	
C14	1.13766 (15)	0.61769 (6)	0.44643 (13)	0.0196 (3)	
C15	1.22967 (16)	0.65118 (6)	0.48600 (14)	0.0253 (3)	
H15	1.278170	0.667819	0.438136	0.030*	
C16	1.25166 (18)	0.66064 (6)	0.59418 (15)	0.0285 (4)	
H16	1.315062	0.683484	0.620127	0.034*	
C17	1.18063 (18)	0.63659 (7)	0.66429 (14)	0.0294 (4)	
H17	1.194008	0.643399	0.738286	0.035*	
C18	1.09024 (18)	0.60266 (7)	0.62648 (14)	0.0304 (4)	
H18	1.042379	0.585924	0.674608	0.036*	
C19	1.06950 (17)	0.59308 (6)	0.51769 (13)	0.0244 (3)	
H19	1.008209	0.569513	0.492122	0.029*	
N21	0.79029 (12)	0.64112 (4)	0.38130 (10)	0.0164 (2)	
C21	0.66912 (15)	0.61953 (5)	0.41439 (12)	0.0171 (3)	
H21	0.693882	0.590491	0.455573	0.020*	
C22	0.56390 (15)	0.60807 (5)	0.32236 (12)	0.0164 (3)	
O22	0.48835 (11)	0.57497 (4)	0.32883 (8)	0.0190 (2)	
C23	0.61858 (16)	0.65603 (6)	0.48916 (13)	0.0222 (3)	
H23A	0.553446	0.677207	0.449635	0.027*	
H23B	0.576450	0.640922	0.547253	0.027*	
C24	0.74280 (17)	0.68210 (7)	0.53261 (14)	0.0264 (4)	
H24	0.721672	0.713795	0.558788	0.032*	
F24	0.81264 (11)	0.65515 (5)	0.61320 (8)	0.0355 (3)	
C25	0.82585 (16)	0.68455 (6)	0.44015 (13)	0.0221 (3)	
H25A	0.802451	0.712231	0.395259	0.026*	
H25B	0.921944	0.685471	0.465593	0.026*	
N31	0.55629 (12)	0.63611 (5)	0.23661 (10)	0.0174 (2)	
H31	0.609437	0.660341	0.236410	0.021*	
C31	0.46039 (15)	0.62662 (5)	0.14377 (12)	0.0177 (3)	
H31A	0.368873	0.632062	0.163798	0.021*	
C32	0.47066 (15)	0.57592 (5)	0.10836 (11)	0.0176 (3)	
O32	0.37026 (11)	0.55339 (4)	0.07621 (9)	0.0230 (2)	
C33	0.48421 (16)	0.66028 (6)	0.05265 (12)	0.0206 (3)	
H33	0.578947	0.657215	0.038293	0.025*	
C34	0.3951 (2)	0.64805 (7)	−0.04927 (14)	0.0365 (4)	
H34A	0.410762	0.615659	−0.068749	0.055*	
H34B	0.301735	0.651964	−0.037470	0.055*	
H34C	0.415658	0.668682	−0.106959	0.055*	
C35	0.46008 (19)	0.71061 (6)	0.08311 (14)	0.0279 (4)	
H35A	0.517319	0.718395	0.148495	0.042*	
H35B	0.480667	0.731270	0.025481	0.042*	
H35C	0.366744	0.714553	0.094971	0.042*	
N41	0.59407 (13)	0.55876 (5)	0.10842 (10)	0.0179 (3)	
H41	0.662919	0.577019	0.125652	0.022*	
C41	0.61575 (15)	0.51035 (5)	0.08037 (12)	0.0182 (3)	
H41A	0.537394	0.499986	0.030664	0.022*	
C42	0.62647 (15)	0.47832 (6)	0.17797 (12)	0.0176 (3)	
O42	0.56513 (11)	0.44117 (4)	0.17630 (9)	0.0219 (2)	
C43	0.73975 (16)	0.50583 (6)	0.02176 (12)	0.0205 (3)	
H43A	0.816942	0.518789	0.067387	0.025*	
H43B	0.726877	0.524739	−0.044091	0.025*	
C44	0.77150 (16)	0.45562 (6)	−0.00784 (13)	0.0223 (3)	
H44	0.791106	0.437464	0.059617	0.027*	
C45	0.89539 (17)	0.45470 (7)	−0.06642 (15)	0.0292 (4)	
H45A	0.969281	0.469672	−0.022382	0.044*	
H45B	0.918882	0.422358	−0.080416	0.044*	
H45C	0.877517	0.471472	−0.134041	0.044*	
C46	0.65390 (17)	0.43247 (6)	−0.07443 (14)	0.0264 (3)	
H46A	0.575668	0.433464	−0.035353	0.040*	
H46B	0.634678	0.449124	−0.142094	0.040*	
H46C	0.676044	0.400010	−0.088470	0.040*	
N51	0.70908 (13)	0.49181 (5)	0.26313 (10)	0.0181 (3)	
H51	0.744760	0.519773	0.265567	0.022*	
C51	0.73863 (15)	0.45960 (6)	0.35103 (13)	0.0191 (3)	
H51A	0.771692	0.430025	0.321616	0.023*	
C52	0.61595 (15)	0.44790 (5)	0.40613 (12)	0.0168 (3)	
O52	0.60186 (11)	0.40807 (4)	0.44008 (9)	0.0212 (2)	
C53	0.84952 (16)	0.47914 (6)	0.43106 (13)	0.0216 (3)	
H53A	0.927063	0.486590	0.392914	0.026*	
H53B	0.818319	0.508587	0.460493	0.026*	
C54	0.89461 (16)	0.44627 (6)	0.52354 (14)	0.0233 (3)	
H54	0.815025	0.438918	0.560710	0.028*	
C55	0.9494 (2)	0.40043 (7)	0.48672 (17)	0.0350 (4)	
H55A	0.882822	0.385489	0.435450	0.053*	
H55B	1.030180	0.406402	0.452806	0.053*	
H55C	0.970653	0.379888	0.548206	0.053*	
C56	0.9957 (2)	0.47046 (7)	0.60338 (16)	0.0329 (4)	
H56A	0.958309	0.499825	0.625939	0.049*	
H56B	1.017130	0.450307	0.665521	0.049*	
H56C	1.076658	0.476821	0.570121	0.049*	
N61	0.53000 (12)	0.48232 (4)	0.41914 (10)	0.0164 (2)	
H61	0.544954	0.510366	0.395133	0.020*	
C61	0.41174 (15)	0.47387 (6)	0.47269 (12)	0.0185 (3)	
H61A	0.353359	0.501929	0.461578	0.022*	
C62	0.33382 (15)	0.43200 (5)	0.42126 (12)	0.0172 (3)	
O62	0.30644 (11)	0.39776 (4)	0.47288 (9)	0.0212 (2)	
C63	0.44372 (17)	0.46661 (6)	0.59353 (12)	0.0228 (3)	
H63A	0.360126	0.460702	0.625056	0.027*	
H63B	0.501168	0.438846	0.606630	0.027*	
C64	0.51300 (17)	0.50829 (6)	0.64742 (12)	0.0218 (3)	
C65	0.64968 (18)	0.50827 (7)	0.67462 (14)	0.0293 (4)	
H65	0.699783	0.481519	0.660147	0.035*	
C66	0.7144 (2)	0.54671 (8)	0.72264 (16)	0.0372 (4)	
H66	0.808136	0.546134	0.740144	0.045*	
C67	0.6433 (2)	0.58569 (7)	0.74503 (15)	0.0386 (5)	
H67	0.687218	0.611861	0.778859	0.046*	
C68	0.5085 (3)	0.58616 (8)	0.71782 (17)	0.0435 (5)	
H68	0.458904	0.612983	0.732648	0.052*	
C69	0.4433 (2)	0.54798 (7)	0.66884 (16)	0.0355 (4)	
H69	0.349832	0.549127	0.649862	0.043*	
N71	0.29783 (13)	0.43468 (5)	0.31474 (10)	0.0175 (3)	
C71	0.22225 (15)	0.39610 (6)	0.26077 (13)	0.0190 (3)	
H71	0.141460	0.390278	0.297619	0.023*	
C72	0.29742 (15)	0.35035 (5)	0.25423 (12)	0.0180 (3)	
O72	0.23391 (12)	0.31421 (4)	0.24196 (10)	0.0275 (3)	
C73	0.17819 (19)	0.41632 (6)	0.14947 (14)	0.0284 (4)	
H73A	0.244808	0.409769	0.099802	0.034*	
H73B	0.091316	0.403346	0.119601	0.034*	
C74	0.16796 (17)	0.46789 (6)	0.16981 (14)	0.0260 (3)	
H74	0.170880	0.486496	0.103235	0.031*	
F74	0.05056 (10)	0.47617 (4)	0.21698 (11)	0.0390 (3)	
C75	0.28595 (16)	0.47786 (6)	0.25075 (13)	0.0208 (3)	
H75A	0.367147	0.483514	0.215744	0.025*	
H75B	0.269227	0.505128	0.295241	0.025*	
N81	0.43076 (13)	0.35202 (5)	0.25851 (10)	0.0178 (3)	
H81	0.471985	0.379034	0.262275	0.021*	
C81	0.50681 (15)	0.30893 (5)	0.25692 (12)	0.0180 (3)	
H81A	0.460344	0.288411	0.200579	0.022*	
C82	0.51262 (15)	0.28270 (6)	0.36357 (13)	0.0181 (3)	
O82	0.49500 (12)	0.24057 (4)	0.36649 (10)	0.0243 (2)	
C83	0.64772 (15)	0.31843 (6)	0.22692 (12)	0.0197 (3)	
H83	0.688957	0.343534	0.274559	0.024*	
C84	0.64158 (19)	0.33527 (6)	0.11155 (14)	0.0267 (3)	
H84A	0.585370	0.363075	0.102177	0.040*	
H84B	0.731400	0.342859	0.095112	0.040*	
H84C	0.604027	0.310675	0.063512	0.040*	
C85	0.73525 (17)	0.27504 (6)	0.24229 (15)	0.0261 (3)	
H85A	0.738818	0.264424	0.316252	0.039*	
H85B	0.697837	0.250359	0.194438	0.039*	
H85C	0.825211	0.282543	0.226039	0.039*	
N91	0.54605 (13)	0.30876 (5)	0.45172 (10)	0.0188 (3)	
H91	0.550529	0.339233	0.445942	0.023*	
C91	0.57471 (15)	0.28714 (5)	0.55599 (12)	0.0181 (3)	
H91A	0.612792	0.255589	0.545360	0.022*	
C92	0.45133 (15)	0.28084 (6)	0.61433 (12)	0.0172 (3)	
O92	0.43563 (11)	0.24499 (4)	0.66468 (9)	0.0230 (2)	
C93	0.68087 (15)	0.31586 (6)	0.62367 (13)	0.0204 (3)	
H93A	0.761563	0.317136	0.586220	0.025*	
H93B	0.647665	0.348113	0.628694	0.025*	
C94	0.72014 (15)	0.29749 (6)	0.73664 (13)	0.0216 (3)	
H94	0.639612	0.297883	0.775743	0.026*	
C95	0.77299 (17)	0.24771 (6)	0.73656 (14)	0.0251 (3)	
H95A	0.796876	0.237325	0.810094	0.038*	
H95B	0.704251	0.227163	0.701387	0.038*	
H95C	0.851773	0.246688	0.698130	0.038*	
C96	0.82464 (17)	0.33006 (7)	0.79417 (15)	0.0297 (4)	
H96A	0.789340	0.361839	0.793580	0.044*	
H96B	0.846288	0.319665	0.867946	0.044*	
H96C	0.905110	0.329512	0.757760	0.044*	
N101	0.36769 (13)	0.31742 (5)	0.61189 (11)	0.0202 (3)	
H101	0.386984	0.343138	0.579064	0.024*	
C101	0.24628 (15)	0.31498 (6)	0.66261 (13)	0.0203 (3)	
H10A	0.260244	0.291319	0.720831	0.024*	
C102	0.12640 (16)	0.29962 (6)	0.58694 (13)	0.0211 (3)	
O102	0.02025 (12)	0.29105 (5)	0.61682 (10)	0.0308 (3)	
C103	0.21605 (15)	0.36172 (6)	0.71397 (13)	0.0207 (3)	
H10B	0.211779	0.386196	0.658532	0.025*	
H10C	0.127114	0.359707	0.739143	0.025*	
C104	0.31611 (17)	0.37686 (7)	0.80731 (14)	0.0269 (4)	
H104	0.404955	0.380506	0.780837	0.032*	
C105	0.27440 (19)	0.42400 (7)	0.84827 (15)	0.0315 (4)	
H10D	0.266375	0.446561	0.789730	0.047*	
H10E	0.188550	0.420891	0.876460	0.047*	
H10F	0.341489	0.434806	0.904906	0.047*	
C106	0.3291 (3)	0.34142 (9)	0.89682 (19)	0.0533 (7)	
H10G	0.355880	0.311345	0.869520	0.080*	
H10H	0.396347	0.351971	0.953618	0.080*	
H10I	0.243408	0.338056	0.925173	0.080*	
O107	0.15034 (11)	0.29707 (4)	0.48560 (9)	0.0240 (2)	
C107	0.03710 (17)	0.28447 (6)	0.41077 (14)	0.0259 (3)	
H10J	0.063848	0.283455	0.338660	0.039*	
H10K	0.004253	0.253792	0.429387	0.039*	
H10L	−0.033467	0.307639	0.413598	0.039*	

### Boc-(D-Phe-tFPro-Val-Leu-Leu)2-OMe Atomic displacement parameters (Å^2^)

**Table:**

	*U*^11^	*U*^22^	*U*^33^	*U*^12^	*U*^13^	*U*^23^
O1M	0.0289 (6)	0.0416 (8)	0.0257 (6)	0.0140 (6)	0.0026 (5)	0.0022 (5)
C1M	0.0348 (9)	0.0346 (10)	0.0283 (9)	−0.0014 (8)	−0.0009 (7)	−0.0003 (7)
C1	0.0318 (9)	0.0269 (9)	0.0198 (8)	0.0034 (7)	−0.0001 (7)	0.0099 (7)
O1	0.0439 (7)	0.0322 (7)	0.0160 (5)	0.0147 (6)	0.0048 (5)	0.0078 (5)
C2	0.0455 (11)	0.0389 (11)	0.0185 (8)	−0.0016 (9)	0.0033 (7)	0.0076 (7)
C3	0.0431 (11)	0.0338 (10)	0.0330 (10)	0.0004 (9)	−0.0004 (8)	0.0003 (8)
C4	0.0318 (10)	0.0433 (12)	0.0383 (10)	0.0011 (8)	0.0008 (8)	0.0216 (9)
C5	0.0304 (9)	0.0295 (9)	0.0184 (7)	0.0050 (7)	0.0039 (6)	0.0051 (6)
O5	0.0499 (8)	0.0475 (9)	0.0236 (6)	0.0287 (7)	0.0094 (6)	0.0083 (6)
N11	0.0223 (6)	0.0237 (7)	0.0148 (6)	0.0027 (5)	0.0037 (5)	0.0030 (5)
C11	0.0182 (7)	0.0187 (7)	0.0151 (7)	−0.0001 (6)	0.0015 (5)	0.0013 (5)
C12	0.0171 (7)	0.0154 (7)	0.0141 (6)	0.0009 (5)	−0.0013 (5)	0.0019 (5)
O12	0.0202 (5)	0.0164 (5)	0.0215 (5)	−0.0009 (4)	0.0019 (4)	−0.0031 (4)
C13	0.0176 (7)	0.0280 (8)	0.0186 (8)	0.0031 (6)	0.0007 (6)	0.0022 (6)
C14	0.0164 (7)	0.0223 (8)	0.0198 (8)	0.0022 (6)	0.0002 (6)	0.0030 (6)
C15	0.0218 (8)	0.0259 (9)	0.0273 (8)	−0.0042 (6)	−0.0016 (6)	0.0061 (7)
C16	0.0282 (8)	0.0238 (9)	0.0314 (9)	−0.0019 (7)	−0.0062 (7)	−0.0016 (7)
C17	0.0295 (9)	0.0374 (10)	0.0204 (8)	0.0031 (7)	−0.0015 (7)	−0.0048 (7)
C18	0.0264 (9)	0.0441 (11)	0.0209 (8)	−0.0038 (7)	0.0033 (7)	0.0057 (7)
C19	0.0220 (8)	0.0282 (9)	0.0221 (8)	−0.0058 (6)	−0.0014 (6)	0.0045 (6)
N21	0.0166 (6)	0.0155 (6)	0.0170 (6)	−0.0010 (5)	0.0017 (5)	−0.0015 (5)
C21	0.0182 (7)	0.0172 (7)	0.0161 (7)	0.0001 (5)	0.0031 (5)	0.0018 (5)
C22	0.0177 (7)	0.0156 (7)	0.0164 (7)	0.0028 (5)	0.0039 (5)	−0.0015 (5)
O22	0.0205 (5)	0.0174 (5)	0.0193 (5)	−0.0019 (4)	0.0033 (4)	0.0003 (4)
C23	0.0217 (7)	0.0257 (8)	0.0198 (7)	−0.0007 (6)	0.0046 (6)	−0.0051 (6)
C24	0.0252 (8)	0.0292 (9)	0.0248 (8)	0.0001 (7)	0.0032 (6)	−0.0092 (7)
F24	0.0296 (5)	0.0580 (8)	0.0178 (5)	−0.0026 (5)	−0.0020 (4)	−0.0041 (5)
C25	0.0234 (8)	0.0186 (8)	0.0242 (8)	−0.0027 (6)	0.0030 (6)	−0.0067 (6)
N31	0.0187 (6)	0.0154 (6)	0.0176 (6)	−0.0014 (5)	−0.0002 (5)	0.0014 (5)
C31	0.0188 (7)	0.0175 (7)	0.0164 (7)	0.0013 (6)	−0.0002 (5)	0.0008 (6)
C32	0.0205 (7)	0.0186 (7)	0.0137 (6)	−0.0003 (6)	0.0013 (5)	0.0031 (5)
O32	0.0204 (5)	0.0211 (6)	0.0262 (6)	−0.0015 (4)	−0.0025 (4)	−0.0001 (4)
C33	0.0250 (8)	0.0196 (8)	0.0170 (7)	0.0033 (6)	0.0019 (6)	0.0021 (6)
C34	0.0560 (12)	0.0294 (10)	0.0212 (8)	−0.0050 (9)	−0.0084 (8)	0.0054 (7)
C35	0.0390 (10)	0.0208 (9)	0.0238 (8)	0.0055 (7)	0.0030 (7)	0.0035 (6)
N41	0.0188 (6)	0.0167 (6)	0.0182 (6)	−0.0014 (5)	0.0014 (5)	−0.0012 (5)
C41	0.0201 (7)	0.0178 (7)	0.0166 (7)	−0.0003 (6)	0.0021 (5)	−0.0030 (6)
C42	0.0177 (7)	0.0174 (7)	0.0186 (7)	0.0005 (6)	0.0059 (5)	−0.0023 (6)
O42	0.0244 (6)	0.0187 (6)	0.0232 (6)	−0.0043 (4)	0.0052 (4)	−0.0040 (4)
C43	0.0211 (7)	0.0236 (8)	0.0172 (7)	−0.0017 (6)	0.0041 (6)	−0.0017 (6)
C44	0.0224 (8)	0.0250 (8)	0.0200 (7)	0.0007 (6)	0.0048 (6)	−0.0037 (6)
C45	0.0217 (8)	0.0383 (10)	0.0282 (9)	0.0016 (7)	0.0054 (7)	−0.0086 (7)
C46	0.0256 (8)	0.0285 (9)	0.0255 (8)	−0.0018 (7)	0.0051 (7)	−0.0092 (7)
N51	0.0210 (6)	0.0156 (6)	0.0179 (6)	−0.0033 (5)	0.0027 (5)	0.0009 (5)
C51	0.0189 (7)	0.0167 (7)	0.0218 (7)	−0.0007 (6)	0.0032 (6)	0.0019 (6)
C52	0.0173 (7)	0.0166 (7)	0.0157 (7)	−0.0020 (5)	−0.0013 (5)	0.0002 (5)
O52	0.0203 (5)	0.0158 (5)	0.0273 (6)	−0.0015 (4)	0.0022 (4)	0.0046 (4)
C53	0.0192 (7)	0.0218 (8)	0.0237 (8)	−0.0019 (6)	0.0015 (6)	0.0001 (6)
C54	0.0201 (7)	0.0225 (8)	0.0264 (8)	0.0028 (6)	−0.0008 (6)	0.0000 (6)
C55	0.0317 (9)	0.0307 (10)	0.0407 (11)	0.0105 (8)	−0.0046 (8)	−0.0054 (8)
C56	0.0321 (9)	0.0316 (10)	0.0325 (9)	0.0023 (7)	−0.0079 (7)	−0.0015 (8)
N61	0.0193 (6)	0.0143 (6)	0.0158 (6)	−0.0007 (5)	0.0026 (5)	0.0020 (5)
C61	0.0207 (7)	0.0190 (7)	0.0162 (7)	0.0003 (6)	0.0036 (6)	0.0003 (6)
C62	0.0157 (7)	0.0186 (7)	0.0176 (7)	0.0013 (5)	0.0036 (5)	0.0014 (6)
O62	0.0204 (5)	0.0220 (6)	0.0215 (5)	−0.0022 (4)	0.0033 (4)	0.0046 (4)
C63	0.0292 (8)	0.0241 (9)	0.0153 (7)	−0.0023 (6)	0.0039 (6)	−0.0005 (6)
C64	0.0291 (8)	0.0236 (8)	0.0131 (7)	0.0004 (6)	0.0035 (6)	0.0004 (6)
C65	0.0289 (9)	0.0350 (10)	0.0247 (8)	0.0028 (7)	0.0059 (7)	−0.0062 (7)
C66	0.0335 (10)	0.0498 (13)	0.0285 (9)	−0.0112 (9)	0.0042 (8)	−0.0074 (8)
C67	0.0602 (13)	0.0315 (10)	0.0223 (8)	−0.0120 (9)	−0.0034 (8)	−0.0034 (7)
C68	0.0647 (14)	0.0297 (11)	0.0330 (10)	0.0140 (10)	−0.0083 (9)	−0.0101 (8)
C69	0.0367 (10)	0.0371 (11)	0.0303 (9)	0.0119 (8)	−0.0060 (8)	−0.0094 (8)
N71	0.0188 (6)	0.0156 (6)	0.0180 (6)	−0.0006 (5)	0.0015 (5)	0.0009 (5)
C71	0.0168 (7)	0.0193 (7)	0.0206 (7)	−0.0014 (6)	0.0003 (6)	−0.0003 (6)
C72	0.0199 (7)	0.0195 (8)	0.0145 (7)	−0.0017 (6)	0.0013 (5)	−0.0006 (6)
O72	0.0232 (6)	0.0222 (6)	0.0376 (7)	−0.0055 (5)	0.0058 (5)	−0.0064 (5)
C73	0.0314 (9)	0.0267 (9)	0.0244 (8)	0.0003 (7)	−0.0089 (7)	0.0023 (7)
C74	0.0231 (8)	0.0270 (9)	0.0268 (8)	0.0027 (6)	−0.0020 (7)	0.0041 (7)
F74	0.0208 (5)	0.0378 (6)	0.0583 (7)	0.0086 (4)	0.0034 (5)	0.0083 (5)
C75	0.0227 (8)	0.0192 (8)	0.0200 (7)	0.0016 (6)	0.0000 (6)	0.0045 (6)
N81	0.0174 (6)	0.0147 (6)	0.0209 (6)	−0.0009 (5)	0.0006 (5)	−0.0002 (5)
C81	0.0182 (7)	0.0157 (7)	0.0199 (7)	−0.0009 (5)	0.0021 (6)	−0.0014 (6)
C82	0.0146 (6)	0.0167 (7)	0.0235 (7)	0.0000 (5)	0.0042 (5)	0.0008 (6)
O82	0.0278 (6)	0.0162 (6)	0.0286 (6)	−0.0028 (4)	0.0027 (5)	0.0017 (5)
C83	0.0199 (7)	0.0190 (8)	0.0208 (7)	−0.0017 (6)	0.0048 (6)	−0.0023 (6)
C84	0.0335 (9)	0.0247 (8)	0.0235 (8)	−0.0001 (7)	0.0095 (7)	−0.0007 (6)
C85	0.0226 (8)	0.0255 (9)	0.0310 (9)	0.0032 (6)	0.0067 (7)	0.0009 (7)
N91	0.0227 (6)	0.0141 (6)	0.0203 (6)	0.0002 (5)	0.0053 (5)	0.0019 (5)
C91	0.0169 (7)	0.0170 (7)	0.0209 (7)	0.0016 (6)	0.0036 (6)	0.0011 (6)
C92	0.0162 (7)	0.0179 (7)	0.0171 (7)	−0.0016 (5)	−0.0004 (5)	0.0008 (6)
O92	0.0230 (5)	0.0206 (6)	0.0259 (6)	0.0004 (4)	0.0048 (4)	0.0061 (5)
C93	0.0172 (7)	0.0193 (8)	0.0252 (8)	−0.0003 (6)	0.0038 (6)	−0.0003 (6)
C94	0.0164 (7)	0.0266 (8)	0.0221 (8)	0.0017 (6)	0.0037 (6)	−0.0029 (6)
C95	0.0224 (7)	0.0282 (9)	0.0244 (8)	0.0033 (6)	0.0022 (6)	0.0031 (7)
C96	0.0230 (8)	0.0361 (10)	0.0298 (9)	0.0001 (7)	0.0022 (7)	−0.0109 (7)
N101	0.0171 (6)	0.0207 (7)	0.0235 (7)	0.0020 (5)	0.0057 (5)	0.0052 (5)
C101	0.0163 (7)	0.0230 (8)	0.0220 (7)	0.0016 (6)	0.0039 (6)	0.0035 (6)
C102	0.0199 (7)	0.0194 (8)	0.0244 (8)	0.0010 (6)	0.0045 (6)	0.0006 (6)
O102	0.0199 (6)	0.0418 (8)	0.0316 (6)	−0.0046 (5)	0.0072 (5)	−0.0049 (6)
C103	0.0166 (7)	0.0248 (8)	0.0209 (7)	0.0020 (6)	0.0024 (6)	0.0010 (6)
C104	0.0189 (7)	0.0336 (9)	0.0275 (9)	−0.0006 (7)	−0.0006 (6)	−0.0033 (7)
C105	0.0292 (9)	0.0383 (11)	0.0273 (9)	−0.0036 (8)	0.0051 (7)	−0.0060 (7)
C106	0.0786 (18)	0.0422 (13)	0.0323 (11)	0.0032 (12)	−0.0229 (11)	0.0042 (9)
O107	0.0223 (5)	0.0272 (6)	0.0228 (6)	−0.0026 (5)	0.0036 (4)	−0.0019 (5)
C107	0.0255 (8)	0.0266 (9)	0.0248 (8)	−0.0020 (7)	−0.0008 (6)	−0.0020 (7)

### Boc-(D-Phe-tFPro-Val-Leu-Leu)2-OMe Geometric parameters (Å, º)

**Table:**

O1M—C1M	1.416 (2)	C54—C55	1.522 (2)
O1M—H1M	0.8400	C54—H54	1.0000
C1M—H1M1	0.9800	C55—H55A	0.9800
C1M—H1M2	0.9800	C55—H55B	0.9800
C1M—H1M3	0.9800	C55—H55C	0.9800
C1—O1	1.4740 (19)	C56—H56A	0.9800
C1—C4	1.510 (3)	C56—H56B	0.9800
C1—C2	1.521 (3)	C56—H56C	0.9800
C1—C3	1.518 (3)	N61—C61	1.4607 (19)
O1—C5	1.349 (2)	N61—H61	0.8800
C2—H2A	0.9800	C61—C63	1.539 (2)
C2—H2B	0.9800	C61—C62	1.541 (2)
C2—H2C	0.9800	C61—H61A	1.0000
C3—H3A	0.9800	C62—O62	1.229 (2)
C3—H3B	0.9800	C62—N71	1.356 (2)
C3—H3C	0.9800	C63—C64	1.510 (2)
C4—H4A	0.9800	C63—H63A	0.9900
C4—H4B	0.9800	C63—H63B	0.9900
C4—H4C	0.9800	C64—C69	1.384 (3)
C5—O5	1.218 (2)	C64—C65	1.388 (3)
C5—N11	1.347 (2)	C65—C66	1.388 (3)
N11—C11	1.4479 (19)	C65—H65	0.9500
N11—H11	0.8800	C66—C67	1.377 (3)
C11—C12	1.534 (2)	C66—H66	0.9500
C11—C13	1.542 (2)	C67—C68	1.370 (3)
C11—H11A	1.0000	C67—H67	0.9500
C12—O12	1.2366 (19)	C68—C69	1.389 (3)
C12—N21	1.340 (2)	C68—H68	0.9500
C13—C14	1.511 (2)	C69—H69	0.9500
C13—H13A	0.9900	N71—C71	1.470 (2)
C13—H13B	0.9900	N71—C75	1.478 (2)
C14—C19	1.388 (2)	C71—C72	1.526 (2)
C14—C15	1.392 (2)	C71—C73	1.541 (2)
C15—C16	1.387 (3)	C71—H71	1.0000
C15—H15	0.9500	C72—O72	1.222 (2)
C16—C17	1.387 (3)	C72—N81	1.346 (2)
C16—H16	0.9500	C73—C74	1.509 (3)
C17—C18	1.385 (3)	C73—H73A	0.9900
C17—H17	0.9500	C73—H73B	0.9900
C18—C19	1.395 (2)	C74—F74	1.409 (2)
C18—H18	0.9500	C74—C75	1.510 (2)
C19—H19	0.9500	C74—H74	1.0000
N21—C25	1.4760 (19)	C75—H75A	0.9900
N21—C21	1.4768 (19)	C75—H75B	0.9900
C21—C22	1.522 (2)	N81—C81	1.459 (2)
C21—C23	1.537 (2)	N81—H81	0.8800
C21—H21	1.0000	C81—C82	1.539 (2)
C22—O22	1.2289 (19)	C81—C83	1.541 (2)
C22—N31	1.345 (2)	C81—H81A	1.0000
C23—C24	1.513 (2)	C82—O82	1.225 (2)
C23—H23A	0.9900	C82—N91	1.353 (2)
C23—H23B	0.9900	C83—C85	1.529 (2)
C24—F24	1.405 (2)	C83—C84	1.531 (2)
C24—C25	1.516 (2)	C83—H83	1.0000
C24—H24	1.0000	C84—H84A	0.9800
C25—H25A	0.9900	C84—H84B	0.9800
C25—H25B	0.9900	C84—H84C	0.9800
N31—C31	1.4616 (19)	C85—H85A	0.9800
N31—H31	0.8800	C85—H85B	0.9800
C31—C32	1.531 (2)	C85—H85C	0.9800
C31—C33	1.543 (2)	N91—C91	1.456 (2)
C31—H31A	1.0000	N91—H91	0.8800
C32—O32	1.235 (2)	C91—C92	1.531 (2)
C32—N41	1.343 (2)	C91—C93	1.536 (2)
C33—C35	1.523 (2)	C91—H91A	1.0000
C33—C34	1.528 (2)	C92—O92	1.230 (2)
C33—H33	1.0000	C92—N101	1.348 (2)
C34—H34A	0.9800	C93—C94	1.532 (2)
C34—H34B	0.9800	C93—H93A	0.9900
C34—H34C	0.9800	C93—H93B	0.9900
C35—H35A	0.9800	C94—C95	1.527 (2)
C35—H35B	0.9800	C94—C96	1.533 (2)
C35—H35C	0.9800	C94—H94	1.0000
N41—C41	1.458 (2)	C95—H95A	0.9800
N41—H41	0.8800	C95—H95B	0.9800
C41—C42	1.533 (2)	C95—H95C	0.9800
C41—C43	1.534 (2)	C96—H96A	0.9800
C41—H41A	1.0000	C96—H96B	0.9800
C42—O42	1.234 (2)	C96—H96C	0.9800
C42—N51	1.343 (2)	N101—C101	1.4508 (19)
C43—C44	1.533 (2)	N101—H101	0.8800
C43—H43A	0.9900	C101—C102	1.523 (2)
C43—H43B	0.9900	C101—C103	1.537 (2)
C44—C45	1.527 (2)	C101—H10A	1.0000
C44—C46	1.530 (2)	C102—O102	1.204 (2)
C44—H44	1.0000	C102—O107	1.332 (2)
C45—H45A	0.9800	C103—C104	1.529 (2)
C45—H45B	0.9800	C103—H10B	0.9900
C45—H45C	0.9800	C103—H10C	0.9900
C46—H46A	0.9800	C104—C106	1.516 (3)
C46—H46B	0.9800	C104—C105	1.526 (3)
C46—H46C	0.9800	C104—H104	1.0000
N51—C51	1.451 (2)	C105—H10D	0.9800
N51—H51	0.8800	C105—H10E	0.9800
C51—C52	1.528 (2)	C105—H10F	0.9800
C51—C53	1.530 (2)	C106—H10G	0.9800
C51—H51A	1.0000	C106—H10H	0.9800
C52—O52	1.2360 (19)	C106—H10I	0.9800
C52—N61	1.339 (2)	O107—C107	1.447 (2)
C53—C54	1.532 (2)	C107—H10J	0.9800
C53—H53A	0.9900	C107—H10K	0.9800
C53—H53B	0.9900	C107—H10L	0.9800
C54—C56	1.521 (2)		
			
C1M—O1M—H1M	109.5	C55—C54—H54	107.6
O1M—C1M—H1M1	109.5	C53—C54—H54	107.6
O1M—C1M—H1M2	109.5	C54—C55—H55A	109.5
H1M1—C1M—H1M2	109.5	C54—C55—H55B	109.5
O1M—C1M—H1M3	109.5	H55A—C55—H55B	109.5
H1M1—C1M—H1M3	109.5	C54—C55—H55C	109.5
H1M2—C1M—H1M3	109.5	H55A—C55—H55C	109.5
O1—C1—C4	110.92 (15)	H55B—C55—H55C	109.5
O1—C1—C2	102.03 (14)	C54—C56—H56A	109.5
C4—C1—C2	110.79 (16)	C54—C56—H56B	109.5
O1—C1—C3	109.70 (14)	H56A—C56—H56B	109.5
C4—C1—C3	112.45 (17)	C54—C56—H56C	109.5
C2—C1—C3	110.47 (16)	H56A—C56—H56C	109.5
C5—O1—C1	121.01 (14)	H56B—C56—H56C	109.5
C1—C2—H2A	109.5	C52—N61—C61	120.81 (13)
C1—C2—H2B	109.5	C52—N61—H61	119.6
H2A—C2—H2B	109.5	C61—N61—H61	119.6
C1—C2—H2C	109.5	N61—C61—C63	112.99 (13)
H2A—C2—H2C	109.5	N61—C61—C62	109.97 (12)
H2B—C2—H2C	109.5	C63—C61—C62	110.67 (13)
C1—C3—H3A	109.5	N61—C61—H61A	107.7
C1—C3—H3B	109.5	C63—C61—H61A	107.7
H3A—C3—H3B	109.5	C62—C61—H61A	107.7
C1—C3—H3C	109.5	O62—C62—N71	121.38 (14)
H3A—C3—H3C	109.5	O62—C62—C61	122.41 (14)
H3B—C3—H3C	109.5	N71—C62—C61	116.21 (13)
C1—C4—H4A	109.5	C64—C63—C61	112.02 (13)
C1—C4—H4B	109.5	C64—C63—H63A	109.2
H4A—C4—H4B	109.5	C61—C63—H63A	109.2
C1—C4—H4C	109.5	C64—C63—H63B	109.2
H4A—C4—H4C	109.5	C61—C63—H63B	109.2
H4B—C4—H4C	109.5	H63A—C63—H63B	107.9
O5—C5—N11	124.00 (15)	C69—C64—C65	117.92 (17)
O5—C5—O1	126.56 (16)	C69—C64—C63	121.34 (16)
N11—C5—O1	109.44 (15)	C65—C64—C63	120.72 (16)
C5—N11—C11	119.41 (13)	C66—C65—C64	121.09 (18)
C5—N11—H11	120.3	C66—C65—H65	119.5
C11—N11—H11	120.3	C64—C65—H65	119.5
N11—C11—C12	109.91 (12)	C67—C66—C65	120.31 (19)
N11—C11—C13	109.16 (12)	C67—C66—H66	119.8
C12—C11—C13	109.22 (12)	C65—C66—H66	119.8
N11—C11—H11A	109.5	C68—C67—C66	119.04 (19)
C12—C11—H11A	109.5	C68—C67—H67	120.5
C13—C11—H11A	109.5	C66—C67—H67	120.5
O12—C12—N21	121.30 (14)	C67—C68—C69	120.95 (19)
O12—C12—C11	119.23 (13)	C67—C68—H68	119.5
N21—C12—C11	119.46 (13)	C69—C68—H68	119.5
C14—C13—C11	111.19 (13)	C64—C69—C68	120.68 (19)
C14—C13—H13A	109.4	C64—C69—H69	119.7
C11—C13—H13A	109.4	C68—C69—H69	119.7
C14—C13—H13B	109.4	C62—N71—C71	119.02 (13)
C11—C13—H13B	109.4	C62—N71—C75	125.92 (13)
H13A—C13—H13B	108.0	C71—N71—C75	111.89 (12)
C19—C14—C15	118.71 (15)	N71—C71—C72	115.97 (13)
C19—C14—C13	121.21 (15)	N71—C71—C73	102.79 (13)
C15—C14—C13	119.96 (15)	C72—C71—C73	111.72 (13)
C14—C15—C16	121.05 (16)	N71—C71—H71	108.7
C14—C15—H15	119.5	C72—C71—H71	108.7
C16—C15—H15	119.5	C73—C71—H71	108.7
C17—C16—C15	119.67 (17)	O72—C72—N81	123.13 (15)
C17—C16—H16	120.2	O72—C72—C71	118.70 (14)
C15—C16—H16	120.2	N81—C72—C71	118.12 (13)
C16—C17—C18	120.03 (16)	C74—C73—C71	103.48 (14)
C16—C17—H17	120.0	C74—C73—H73A	111.1
C18—C17—H17	120.0	C71—C73—H73A	111.1
C17—C18—C19	119.95 (16)	C74—C73—H73B	111.1
C17—C18—H18	120.0	C71—C73—H73B	111.1
C19—C18—H18	120.0	H73A—C73—H73B	109.0
C14—C19—C18	120.56 (16)	F74—C74—C75	108.79 (14)
C14—C19—H19	119.7	F74—C74—C73	108.38 (15)
C18—C19—H19	119.7	C75—C74—C73	103.72 (13)
C12—N21—C25	127.59 (13)	F74—C74—H74	111.9
C12—N21—C21	120.16 (13)	C75—C74—H74	111.9
C25—N21—C21	112.05 (12)	C73—C74—H74	111.9
N21—C21—C22	114.00 (12)	N71—C75—C74	102.76 (13)
N21—C21—C23	103.43 (12)	N71—C75—H75A	111.2
C22—C21—C23	111.37 (12)	C74—C75—H75A	111.2
N21—C21—H21	109.3	N71—C75—H75B	111.2
C22—C21—H21	109.3	C74—C75—H75B	111.2
C23—C21—H21	109.3	H75A—C75—H75B	109.1
O22—C22—N31	122.50 (14)	C72—N81—C81	119.85 (13)
O22—C22—C21	120.24 (13)	C72—N81—H81	120.1
N31—C22—C21	117.24 (13)	C81—N81—H81	120.1
C24—C23—C21	103.89 (13)	N81—C81—C82	111.78 (12)
C24—C23—H23A	111.0	N81—C81—C83	110.97 (12)
C21—C23—H23A	111.0	C82—C81—C83	110.92 (12)
C24—C23—H23B	111.0	N81—C81—H81A	107.7
C21—C23—H23B	111.0	C82—C81—H81A	107.7
H23A—C23—H23B	109.0	C83—C81—H81A	107.7
F24—C24—C23	108.67 (15)	O82—C82—N91	123.07 (15)
F24—C24—C25	107.85 (13)	O82—C82—C81	121.25 (14)
C23—C24—C25	104.71 (13)	N91—C82—C81	115.56 (13)
F24—C24—H24	111.8	C85—C83—C84	109.83 (13)
C23—C24—H24	111.8	C85—C83—C81	111.33 (13)
C25—C24—H24	111.8	C84—C83—C81	110.55 (13)
N21—C25—C24	102.96 (13)	C85—C83—H83	108.3
N21—C25—H25A	111.2	C84—C83—H83	108.3
C24—C25—H25A	111.2	C81—C83—H83	108.3
N21—C25—H25B	111.2	C83—C84—H84A	109.5
C24—C25—H25B	111.2	C83—C84—H84B	109.5
H25A—C25—H25B	109.1	H84A—C84—H84B	109.5
C22—N31—C31	120.49 (13)	C83—C84—H84C	109.5
C22—N31—H31	119.8	H84A—C84—H84C	109.5
C31—N31—H31	119.8	H84B—C84—H84C	109.5
N31—C31—C32	110.57 (12)	C83—C85—H85A	109.5
N31—C31—C33	109.66 (12)	C83—C85—H85B	109.5
C32—C31—C33	110.88 (12)	H85A—C85—H85B	109.5
N31—C31—H31A	108.6	C83—C85—H85C	109.5
C32—C31—H31A	108.6	H85A—C85—H85C	109.5
C33—C31—H31A	108.6	H85B—C85—H85C	109.5
O32—C32—N41	122.61 (15)	C82—N91—C91	121.00 (13)
O32—C32—C31	120.97 (14)	C82—N91—H91	119.5
N41—C32—C31	116.30 (13)	C91—N91—H91	119.5
C35—C33—C34	109.59 (14)	N91—C91—C92	113.34 (12)
C35—C33—C31	111.29 (13)	N91—C91—C93	109.28 (13)
C34—C33—C31	110.96 (14)	C92—C91—C93	110.67 (12)
C35—C33—H33	108.3	N91—C91—H91A	107.8
C34—C33—H33	108.3	C92—C91—H91A	107.8
C31—C33—H33	108.3	C93—C91—H91A	107.8
C33—C34—H34A	109.5	O92—C92—N101	123.16 (14)
C33—C34—H34B	109.5	O92—C92—C91	120.53 (14)
H34A—C34—H34B	109.5	N101—C92—C91	116.20 (13)
C33—C34—H34C	109.5	C94—C93—C91	115.18 (13)
H34A—C34—H34C	109.5	C94—C93—H93A	108.5
H34B—C34—H34C	109.5	C91—C93—H93A	108.5
C33—C35—H35A	109.5	C94—C93—H93B	108.5
C33—C35—H35B	109.5	C91—C93—H93B	108.5
H35A—C35—H35B	109.5	H93A—C93—H93B	107.5
C33—C35—H35C	109.5	C95—C94—C93	112.09 (13)
H35A—C35—H35C	109.5	C95—C94—C96	110.42 (14)
H35B—C35—H35C	109.5	C93—C94—C96	108.92 (14)
C32—N41—C41	121.05 (13)	C95—C94—H94	108.4
C32—N41—H41	119.5	C93—C94—H94	108.4
C41—N41—H41	119.5	C96—C94—H94	108.4
N41—C41—C42	112.08 (12)	C94—C95—H95A	109.5
N41—C41—C43	110.61 (13)	C94—C95—H95B	109.5
C42—C41—C43	110.52 (12)	H95A—C95—H95B	109.5
N41—C41—H41A	107.8	C94—C95—H95C	109.5
C42—C41—H41A	107.8	H95A—C95—H95C	109.5
C43—C41—H41A	107.8	H95B—C95—H95C	109.5
O42—C42—N51	121.99 (15)	C94—C96—H96A	109.5
O42—C42—C41	121.04 (14)	C94—C96—H96B	109.5
N51—C42—C41	116.93 (13)	H96A—C96—H96B	109.5
C41—C43—C44	113.77 (13)	C94—C96—H96C	109.5
C41—C43—H43A	108.8	H96A—C96—H96C	109.5
C44—C43—H43A	108.8	H96B—C96—H96C	109.5
C41—C43—H43B	108.8	C92—N101—C101	120.92 (13)
C44—C43—H43B	108.8	C92—N101—H101	119.5
H43A—C43—H43B	107.7	C101—N101—H101	119.5
C45—C44—C46	110.90 (14)	N101—C101—C102	113.04 (13)
C45—C44—C43	109.93 (14)	N101—C101—C103	111.32 (13)
C46—C44—C43	111.80 (14)	C102—C101—C103	109.61 (13)
C45—C44—H44	108.0	N101—C101—H10A	107.5
C46—C44—H44	108.0	C102—C101—H10A	107.5
C43—C44—H44	108.0	C103—C101—H10A	107.5
C44—C45—H45A	109.5	O102—C102—O107	123.78 (15)
C44—C45—H45B	109.5	O102—C102—C101	122.58 (15)
H45A—C45—H45B	109.5	O107—C102—C101	113.63 (13)
C44—C45—H45C	109.5	C104—C103—C101	115.35 (14)
H45A—C45—H45C	109.5	C104—C103—H10B	108.4
H45B—C45—H45C	109.5	C101—C103—H10B	108.4
C44—C46—H46A	109.5	C104—C103—H10C	108.4
C44—C46—H46B	109.5	C101—C103—H10C	108.4
H46A—C46—H46B	109.5	H10B—C103—H10C	107.5
C44—C46—H46C	109.5	C106—C104—C105	110.28 (17)
H46A—C46—H46C	109.5	C106—C104—C103	112.25 (16)
H46B—C46—H46C	109.5	C105—C104—C103	109.23 (14)
C42—N51—C51	119.05 (13)	C106—C104—H104	108.3
C42—N51—H51	120.5	C105—C104—H104	108.3
C51—N51—H51	120.5	C103—C104—H104	108.3
N51—C51—C52	112.40 (12)	C104—C105—H10D	109.5
N51—C51—C53	110.16 (13)	C104—C105—H10E	109.5
C52—C51—C53	110.65 (13)	H10D—C105—H10E	109.5
N51—C51—H51A	107.8	C104—C105—H10F	109.5
C52—C51—H51A	107.8	H10D—C105—H10F	109.5
C53—C51—H51A	107.8	H10E—C105—H10F	109.5
O52—C52—N61	122.59 (14)	C104—C106—H10G	109.5
O52—C52—C51	119.56 (14)	C104—C106—H10H	109.5
N61—C52—C51	117.80 (13)	H10G—C106—H10H	109.5
C54—C53—C51	114.29 (14)	C104—C106—H10I	109.5
C54—C53—H53A	108.7	H10G—C106—H10I	109.5
C51—C53—H53A	108.7	H10H—C106—H10I	109.5
C54—C53—H53B	108.7	C102—O107—C107	115.16 (13)
C51—C53—H53B	108.7	O107—C107—H10J	109.5
H53A—C53—H53B	107.6	O107—C107—H10K	109.5
C56—C54—C55	110.89 (15)	H10J—C107—H10K	109.5
C56—C54—C53	110.16 (15)	O107—C107—H10L	109.5
C55—C54—C53	112.81 (15)	H10J—C107—H10L	109.5
C56—C54—H54	107.6	H10K—C107—H10L	109.5
			
C4—C1—O1—C5	−67.7 (2)	C51—C53—C54—C55	60.54 (19)
C2—C1—O1—C5	174.22 (16)	O52—C52—N61—C61	−1.6 (2)
C3—C1—O1—C5	57.1 (2)	C51—C52—N61—C61	−178.98 (13)
C1—O1—C5—O5	16.7 (3)	C52—N61—C61—C63	71.94 (18)
C1—O1—C5—N11	−163.82 (15)	C52—N61—C61—C62	−52.27 (17)
O5—C5—N11—C11	−6.9 (3)	N61—C61—C62—O62	123.82 (15)
O1—C5—N11—C11	173.63 (14)	C63—C61—C62—O62	−1.7 (2)
C5—N11—C11—C12	74.94 (18)	N61—C61—C62—N71	−55.49 (17)
C5—N11—C11—C13	−165.29 (15)	C63—C61—C62—N71	178.96 (13)
N11—C11—C12—O12	50.57 (18)	N61—C61—C63—C64	59.55 (18)
C13—C11—C12—O12	−69.16 (17)	C62—C61—C63—C64	−176.62 (13)
N11—C11—C12—N21	−129.12 (14)	C61—C63—C64—C69	77.8 (2)
C13—C11—C12—N21	111.15 (15)	C61—C63—C64—C65	−100.59 (18)
N11—C11—C13—C14	164.46 (13)	C69—C64—C65—C66	0.5 (3)
C12—C11—C13—C14	−75.34 (16)	C63—C64—C65—C66	178.97 (16)
C11—C13—C14—C19	84.19 (19)	C64—C65—C66—C67	0.5 (3)
C11—C13—C14—C15	−91.77 (18)	C65—C66—C67—C68	−0.9 (3)
C19—C14—C15—C16	−1.2 (3)	C66—C67—C68—C69	0.3 (3)
C13—C14—C15—C16	174.85 (16)	C65—C64—C69—C68	−1.1 (3)
C14—C15—C16—C17	−0.3 (3)	C63—C64—C69—C68	−179.56 (18)
C15—C16—C17—C18	1.3 (3)	C67—C68—C69—C64	0.7 (3)
C16—C17—C18—C19	−0.8 (3)	O62—C62—N71—C71	1.2 (2)
C15—C14—C19—C18	1.7 (3)	C61—C62—N71—C71	−179.47 (12)
C13—C14—C19—C18	−174.27 (16)	O62—C62—N71—C75	159.38 (14)
C17—C18—C19—C14	−0.8 (3)	C61—C62—N71—C75	−21.3 (2)
O12—C12—N21—C25	177.70 (14)	C62—N71—C71—C72	−68.58 (18)
C11—C12—N21—C25	−2.6 (2)	C75—N71—C71—C72	130.36 (14)
O12—C12—N21—C21	3.3 (2)	C62—N71—C71—C73	169.24 (14)
C11—C12—N21—C21	−177.06 (13)	C75—N71—C71—C73	8.17 (16)
C12—N21—C21—C22	−56.44 (18)	N71—C71—C72—O72	157.41 (14)
C25—N21—C21—C22	128.32 (14)	C73—C71—C72—O72	−85.27 (18)
C12—N21—C21—C23	−177.53 (13)	N71—C71—C72—N81	−24.9 (2)
C25—N21—C21—C23	7.23 (16)	C73—C71—C72—N81	92.43 (17)
N21—C21—C22—O22	149.83 (14)	N71—C71—C73—C74	−29.13 (16)
C23—C21—C22—O22	−93.60 (17)	C72—C71—C73—C74	−154.14 (14)
N21—C21—C22—N31	−31.60 (19)	C71—C73—C74—F74	−75.89 (16)
C23—C21—C22—N31	84.96 (16)	C71—C73—C74—C75	39.61 (17)
N21—C21—C23—C24	−26.60 (16)	C62—N71—C75—C74	−143.55 (15)
C22—C21—C23—C24	−149.45 (13)	C71—N71—C75—C74	15.93 (16)
C21—C23—C24—F24	−78.57 (15)	F74—C74—C75—N71	81.25 (15)
C21—C23—C24—C25	36.47 (17)	C73—C74—C75—N71	−33.96 (16)
C12—N21—C25—C24	−159.85 (15)	O72—C72—N81—C81	−4.9 (2)
C21—N21—C25—C24	14.95 (16)	C71—C72—N81—C81	177.49 (13)
F24—C24—C25—N21	84.12 (15)	C72—N81—C81—C82	−73.56 (17)
C23—C24—C25—N21	−31.49 (17)	C72—N81—C81—C83	162.04 (13)
O22—C22—N31—C31	−3.2 (2)	N81—C81—C82—O82	135.54 (15)
C21—C22—N31—C31	178.24 (13)	C83—C81—C82—O82	−100.04 (17)
C22—N31—C31—C32	−50.62 (18)	N81—C81—C82—N91	−48.28 (18)
C22—N31—C31—C33	−173.19 (13)	C83—C81—C82—N91	76.14 (16)
N31—C31—C32—O32	141.57 (14)	N81—C81—C83—C85	170.89 (13)
C33—C31—C32—O32	−96.58 (17)	C82—C81—C83—C85	46.01 (17)
N31—C31—C32—N41	−42.26 (17)	N81—C81—C83—C84	−66.74 (16)
C33—C31—C32—N41	79.60 (16)	C82—C81—C83—C84	168.38 (13)
N31—C31—C33—C35	−63.52 (17)	O82—C82—N91—C91	5.5 (2)
C32—C31—C33—C35	174.09 (13)	C81—C82—N91—C91	−170.62 (13)
N31—C31—C33—C34	174.16 (14)	C82—N91—C91—C92	−88.93 (17)
C32—C31—C33—C34	51.78 (18)	C82—N91—C91—C93	147.14 (14)
O32—C32—N41—C41	−6.4 (2)	N91—C91—C92—O92	139.31 (15)
C31—C32—N41—C41	177.47 (13)	C93—C91—C92—O92	−97.52 (17)
C32—N41—C41—C42	−90.60 (16)	N91—C91—C92—N101	−44.39 (19)
C32—N41—C41—C43	145.58 (13)	C93—C91—C92—N101	78.78 (16)
N41—C41—C42—O42	133.46 (15)	N91—C91—C93—C94	179.32 (13)
C43—C41—C42—O42	−102.66 (16)	C92—C91—C93—C94	53.84 (17)
N41—C41—C42—N51	−48.94 (18)	C91—C93—C94—C95	58.34 (17)
C43—C41—C42—N51	74.94 (17)	C91—C93—C94—C96	−179.18 (13)
N41—C41—C43—C44	178.43 (12)	O92—C92—N101—C101	−5.1 (2)
C42—C41—C43—C44	53.71 (17)	C91—C92—N101—C101	178.71 (13)
C41—C43—C44—C45	179.55 (13)	C92—N101—C101—C102	−93.02 (17)
C41—C43—C44—C46	55.94 (18)	C92—N101—C101—C103	143.10 (14)
O42—C42—N51—C51	7.0 (2)	N101—C101—C102—O102	172.30 (15)
C41—C42—N51—C51	−170.57 (13)	C103—C101—C102—O102	−62.9 (2)
C42—N51—C51—C52	−64.51 (18)	N101—C101—C102—O107	−8.2 (2)
C42—N51—C51—C53	171.59 (13)	C103—C101—C102—O107	116.57 (15)
N51—C51—C52—O52	143.48 (14)	N101—C101—C103—C104	−65.50 (18)
C53—C51—C52—O52	−92.89 (17)	C102—C101—C103—C104	168.69 (14)
N51—C51—C52—N61	−39.04 (19)	C101—C103—C104—C106	−58.4 (2)
C53—C51—C52—N61	84.59 (17)	C101—C103—C104—C105	178.95 (14)
N51—C51—C53—C54	−176.21 (13)	O102—C102—O107—C107	1.9 (2)
C52—C51—C53—C54	58.88 (18)	C101—C102—O107—C107	−177.52 (14)
C51—C53—C54—C56	−174.94 (14)		

### Boc-(D-Phe-tFPro-Val-Leu-Leu)2-OMe Hydrogen-bond geometry (Å, º)

**Table:**

*D*—H···*A*	*D*—H	H···*A*	*D*···*A*	*D*—H···*A*
N41—H41···O12	0.88	2.29	2.968 (2)	134
N51—H51···O12	0.88	1.95	2.828 (2)	179
N61—H61···O22	0.88	2.09	2.907 (2)	154
N81—H81···O42	0.88	2.34	3.132 (2)	149
N91—H91···O52	0.88	2.05	2.913 (2)	168
N101—H101···O62	0.88	2.16	2.924 (2)	144
O1*M*—H1*M*···O32	0.84	1.88	2.703 (2)	164
N11—H11···O1*M*^i^	0.88	2.01	2.880 (2)	170

Symmetry code: (i) *x*+1, *y*, *z*.
